# What is the most effective local anesthesia for transrectal ultrasonography-guided biopsy of the prostate? A systematic review and network meta-analysis of 47 randomized clinical trials

**DOI:** 10.1038/s41598-019-41412-w

**Published:** 2019-03-20

**Authors:** Do Kyung Kim, Joo Yong Lee, Jae Hung Jung, Yoon Soo Hah, Kyo Chul Koo, Kwang Suk Lee, Byung Ha Chung, Kang Su Cho

**Affiliations:** 1 Department of Urology, Gangnam Severance Hospital, Urological Science Institute, Yonsei University College of Medicine, Seoul, Republic of Korea; 2 Department of Urology, Severance Hospital, Urological Science Institute, Yonsei University College of Medicine, Seoul, Republic of Korea; 3 Department of Urology, Institute of Evidence Based Medicine, Yonsei University Wonju College of Medicine, Wonju, Republic of Korea

## Abstract

We aimed to compare the effectiveness of various local anesthetic methods for controlling prostate biopsy (PBx) related pain using network meta-analysis. Literature searches were performed on PubMed/Medline, Embase, and Cochrane Library up to March 2018. Forty-seven randomized controlled trials, in which the effectiveness of PBx-related pain was investigated using a visual analogue scale after various local anesthetic methods, were included. The local anesthetic methods included intraprostatic local anesthesia (IPLA), intrarectal local anesthesia (IRLA), intravenous sedation (IVS), periprostatic nerve block (PNB), pelvic plexus block (PPB), and spinal anesthesia (SPA). Eight pairwise meta-analyses and network meta-analyses with 21 comparisons were performed. All modalities, except single use of IPLA and IRLA, were more effective than placebo. Our results demonstrate that PNB + IVS (rank 1) and SPA (rank 2) were the most effective methods for pain control. The followings are in order of PPB + IRLA, PNB + IPLA, PPB, PNB + IRLA, IVS, and PNB. In conclusion, the most effective way to alleviate PBx-related pain appears to be PNB + IVS and SPA. However, a potential increase in medical cost and additional risk of morbidities should be considered. In the current outpatient setting, PPB + IRLA, PNB + IPLA, PPB, PNB + IRLA, and PNB methods are potentially more acceptable options.

## Introduction

The introduction of transrectal ultrasonography (TRUS)-guided prostate biopsy (PBx) in 1989 was a significant development in diagnostic methodology for detection of prostate cancer (PCa). This new method quickly became the gold standard for the detection of PCa and remains the method of choice to the present day^1^. Although many patients can tolerate PBx the procedure can cause severe pain^2^. A random sampling method is used in PBx, so 10 or more core biopsy recommended to increase the detection rate of PCa^3^. However, as the number of core increases to achieve better detection rate, the patient experiences more pain. Severe pain can cause the patient to move during the procedure, which can increase the rate of complications and decrease the number of core that can be taken. These factors can potentially decrease accuracy of the final diagnosis^4,5^. These pitfalls highlight the importance of performing PBx with optimal local anesthesia for patient safety and accurate diagnosis^4^.

Various types of local anesthesia have been used for TRUS-guided PBx, including the most common methods of intrarectal local anesthesia (IRLA) and periprostatic nerve block (PNB)^6^. IRLA has been recommended as a non-invasive alternative to relieve pain during PBx^7^. Periprostatic injection of lidocaine into the neurovascular bundles markedly decreases the discomfort associated with PBx^8^. In addition to IRLA and PNB, intraprostatic local anesthesia (IPLA), spinal anesthesia (SPA; low dose spinal anesthesia or caudal block), intravenous sedation (IVS), and pelvic plexus block (PPB) are used for pain control during PBx. There are many randomized control trials (RCTs) that compare the effects of these anesthetic methods, alone or in combination, with placebo. However, the question of which method is the most effective way to control pain during PBx remains unanswered.

The purpose of the present study was to compare the effectiveness of various local anesthetic methods for controlling PBx-related pain to determine the best approach. To accomplish this, we performed a systematic review of the published literature and network meta-analysis of the available data.

## Results

### Systematic review process

**Figure 1:**
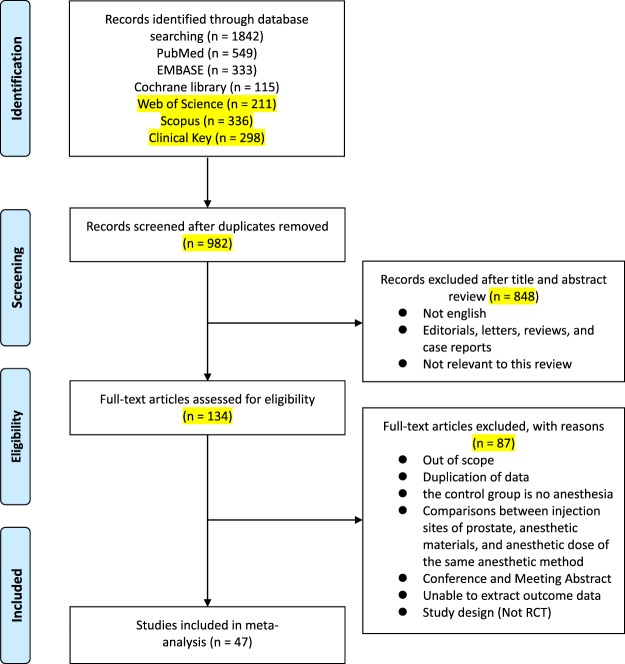
Preferred Reporting Items for Systematic Reviews and Meta-analysis flowchart RCT: randomized controlled trial.

A summary of the analysis results is shown as a PRISMA flow diagram, representing a visual flowchart, in Fig. 1. A total of 1842 articles were found in initial database searches. Of these, 982 articles remained after removing duplicates. After excluding non-English articles, editorials, letters, reviews, case reports, and articles not related to this study by reviewing the titles and abstracts of all available literature, 134 articles remained. Next, a review of full-text articles was performed to evaluate whether they met the inclusion criteria. A total of 47 RCTs^1–5,9–50^ with a total of 5,758 patients were finally included in this study. The characteristics of included studies are displayed in Table 1. All articles were prospective RCTs, and VAS was used to assess pain in all trials. The PNB injection site was the base, with or without apex. The number of PBx core ranged from 6 to 14 across all studies.

**Table 1:** Characteristics of included studies.

First Author, year	Study design	Treatment arms (number of patients)	Anesthetics	Number of prostatic core	Injection Site of PNB	Pain Scale
Adamakis, 2004^9^	RCT	P (40); IRLA (78); PNB (80)	PNB: 2% lidocaine (5 mL) IRLA: Lidocaine–prilocaine cream (10 mL)	10	Base	VAS
Akpınar, 2009^10^	RCT	PNB (40); PPB (40)	PNB: 2% lidocaine (2 mL) PPB: 2% lidocaine (2 mL)	12	Base	VAS
Aktoz, 2009^11^	RCT	PNB (30); IRLA (30); PNB + IRLA (30)	PNB: 0.75% levobupivacaine (3.3 mL) IRLA: Diclofenac sodium suppository (50 mg)	10	Base + Apex	VAS
Alavi, 2001^12^	RCT	PNB(75); IRLA(75)	PNB: 1% lidocaine (10 mL) IRLA: 2% lidocaine gel (10 mL)	6–14	Base	VAS
Anastasi, 2016^13^	RCT	PNB (50); IRLA (50)	PNB: 1% lidocaine (10 mL) IRLA: 1% lidocaine gel (5 mL)	12	NA	VAS
Atta, 2018^14^	RCT	PNB (100); IVS (100); PNB + IVS (100)	PNB: 1% lidocaine (10 mL) IVS: diazepam (5 mg)	10	Base	VAS
Bingqian, 2009^15^	RCT	PNB (100); PNB + IPLA (100)	PNB: 1% lidocaine (10 mL) IPLA: 1% lidocaine (10 mL)	14	Base	VAS
Cam, 2008^16^	RCT	PNB (100); PNB + IPLA (100)	PNB: 2% lidocaine (5 mL) IPLA: 2% lidocaine (5 mL)	12	Base	VAS
Cantiello, 2012^17^	RCT	PPB + IRLA (90); PNB + IRLA (90)	PPB and PNB: 1% lidocaine + 0.75% naropine (2.5 mL) IRLA: 1.5% lidocaine gel (10 mL) + 0.3% nifedipine cream	12	Base	VAS
Cevik, 2002^18^	RCT	P (50); IRLA (50)	IRLA: 2% lidocaine gel (20 mL)	>8		VAS
Chang, 2001^19^	RCT	P (52); IRLA(56)	IRLA: 2% lidocaine gel (10 mL)	>8		VAS
Galosi, 2005^5^	RCT	P(19); IRLA(60)	IRLA: EMLA cream (5mL) and 2.5% lidocaine gel (10 mL)	6–7		VAS
Giannarini, 2009^4^	RCT	PNB (68); IRLA (67); PNB + IRLA (68)	PNB: 1% lidocaine (10 mL) IRLA: Lidocaine–prilocaine cream (5 mL)	10	Base	VAS
Goluza, 2010^20^	RCT	P (80); IRLA(80)	IRLA: Lidocaine suppository (60 mL)	12		VAS
Gurubuz, 2010^21^	RCT	PNB (25); IRLA (25)	PNB: 1% lidocaine (10 mL) IRLA: Lidocaine–prilocaine cream (5mL)	10	Base + Apex	VAS
Hiros, 2010^22^	RCT	PNB (30); IRLA (30)	PNB: 1% lidocaine (10 mL) IRLA: Voltaren Suppository	10	Base + Apex	VAS
Inal, 2004^23^	RCT	P (30); PNB (30)	PNB: 1% lidocaine	6–12	Base	VAS
Ingber, 2010^3^	RCT	P (49); PNB (49)	PNB: 1% lidocaine (5mL)	10	Base	VAS
Izoi, 2012^2^	RCT	PNB (25); IRLA (25)	PNB: 2% lidocaine (5mL) IRLA: 2% lidocaine gel (10 mL)	12	Base	VAS
Jindal, 2014^24^	RCT	IRLA (46); PPB + IRLA (47); PNB + IRLA(46)	PPB and PNB: 2% lidocaine (5mL) IRLA: 2% lidocaine gel (10 mL)	12	Base	VAS
Kravchick, 2004^25^	RCT	PNB (27); IRLA (28)	PNB: 1% lidocaine (5mL) IRLA: 2% lidocaine gel (10 mL)	6	NA	VAS
Kucur, 2015^1^	RCT	PNB + IRLA (50); SPA (50)	PNB: 2% lidocaine (5mL) IRLA: 2% lidocaine gel (10 mL) SPA: 0.5% bupivacaine (0.3mL)	14	Base	VAS
Kumar, 2013^26^	RCT	PNB (50); PNB + IPLA (50)	PNB: 1% lidocaine (10 mL) IPLA: 1% lidocaine (5 mL)	12	Base	VAS
Lee, 2004^28^	RCT	PNB (35); IRLA (37)	PNB: 1% lidocaine (5mL) IRLA: 2% lidocaine gel (10 mL)	8–12	Base	VAS
Lee, 2007^27^	RCT	PNB (49); IPLA (41); PNB + IPLA (62)	PNB: 1% lidocaine (2 mL) IPLA: 1% lidocaine (2 mL)	12	Base	VAS
Leung, 2006^29^	RCT	P (169); IRLA (169)	IRLA: 2% lidocaine gel (10 mL)	10		VAS
Mallick, 2005^30^	RCT	PNB (176); IRLA (180)	PNB: 1% lidocaine (10 mL) IRLA: 2% lidocaine gel (15 mL)	10	Base	VAS
Mazdak, 2018^31^	RCT	PNB + IRLA (36); IVS (35); SPA (35)	PNB: 2% lidocaine (5 mL) IRLA: 2% lidocaine gel (10 mL) IVS: midazolam (25 μg/kg), fentanyl (2 μg/kg), and ketamine (1 mg/kg) SPA: 0.5% bupivacaine (1.5mL)	12	Base	VAS
Nambirajan, 2004^32^	RCT	P (48); PNB (48)	PNB: 1% lidocaine (5 mL)	10	Base + Apex	VAS
Noh, 2010^33^	RCT	PNB (38); PNB + IRLA (36)	PNB: 1% lidocaine (10 mL) IRLA: Lidocaine–prilocaine cream (5 mL)	12	NA	VAS
Obi, 2011^34^	RCT	PNB (25); SPA (25)	PNB: 1% lidocaine (10 mL) SPA: 0.5% bupivacaine (0.5 mL)	6–8	Base + Apex	VAS
Ozden, 2003^35^	RCT	P (25); PNB (25)	PNB: 1% lidocaine (10 mL)	8	Base, Base + Apex	VAS
Raber, 2005^37^	RCT	P (49); IRLA(48)	IRLA: Lidocaine–prilocaine cream (5 mL)	12		VAS
Raber, 2008^36^	RCT	PNB (100); PNB + IRLA (100)	PNB: 2% lidocaine (10 mL) IRLA: Lidocaine–prilocaine cream (5 mL)	14	Base	VAS
Rodriguez, 2003^38^	RCT	PNB (53); IRLA (43)	PNB: 1% lidocaine (10 mL) IRLA: 2% lidocaine gel (10 mL)	6–14	Apex	VAS
Seckiner, 2011^39^	RCT	P (31); PNB (29)	PNB: 2% lidocaine (5 mL)	7–8	Base	VAS
Singh, 2012^40^	RCT	P (49); PNB (46); PNB + IPLA (47)	PNB: 1% lidocaine (10 mL) IPLA: 1% lidocaine (5 mL)	12	Base	VAS
Skriapas, 2011^41^	RCT	PNB (73); PNB + IRLA (74)	PNB: 2% lidocaine (5 mL) IRLA: 2% lidocaine gel (10 mL)	12	NA	VAS
Song, 2006^42^	RCT	P (30); PNB (30); IRLA (30)	PNB: 2% lidocaine (5 mL) IRLA: 2% lidocaine gel (10 mL)	10	Base	VAS
Szlauer, 2008^43^	RCT	PNB (25); PNB + IRLA (25)	PNB: 2% lidocaine (10 mL) IRLA: 2% lidocaine gel (10 mL)	10	Base	VAS
Trucchi, 2005^44^	RCT	PNB (20); IRLA (20)	PNB: 2% lidocaine (10 mL) IRLA: 2% lidocaine gel (10 mL)	10	Base	VAS
Turgut, 2006^50^	RCT	PNB (31); IVS (31)	PNB: 2% lidocaine (10 mL) IVS: midazolam (0.07–0.1 mg/kg)	12	Base	VAS
Vanni, 2004^45^	RCT	P (20); PNB (20)	PNB: 2% lidocaine (10 mL)	10–12	Base	VAS
Wang, 2016^46^	RCT	PNB + IRLA (95); SPA (92)	PNB: 1% lidocaine + 0.5% ropivacaine (5mL) IRLA: 0.3% oxybuprocaine gel (10 mL) SPA: 1.2% lidocaine (20mL)	12	Base	VAS
Wu, 2001^47^	RCT	P (19); PPB (21)	PPB: 1% lidocaine (5mL)	12		VAS
Yun, 2007^48^	RCT	PNB (113); PNB + IRLA (90)	PNB: 1% lidocaine (8mL) IRLA: 2% lidocaine gel (10 mL)	12	Base	VAS
Yurdakul, 2009^49^	RCT	IRLA (25); PNB + IRLA (50)	PNB: 0.25% ropivacaine (5 mL) IRLA: 2% lidocaine gel (10 mL)	10	Base	VAS

**IPLA**: intraprostatic local anesthesia, **IRLA**: intrarectal local anesthesia, IVS: intravenous sedation **PNB**: periprostatic nerve block, **P**: placebo, **PPB**: pelvic plexus block, **RCT**: randomized controlled trial, **SPA**: spinal anesthesia, VAS: visual analog scale.

### Pairwise meta-analysis

#### IRLA vs. Placebo

**Figure 2:**
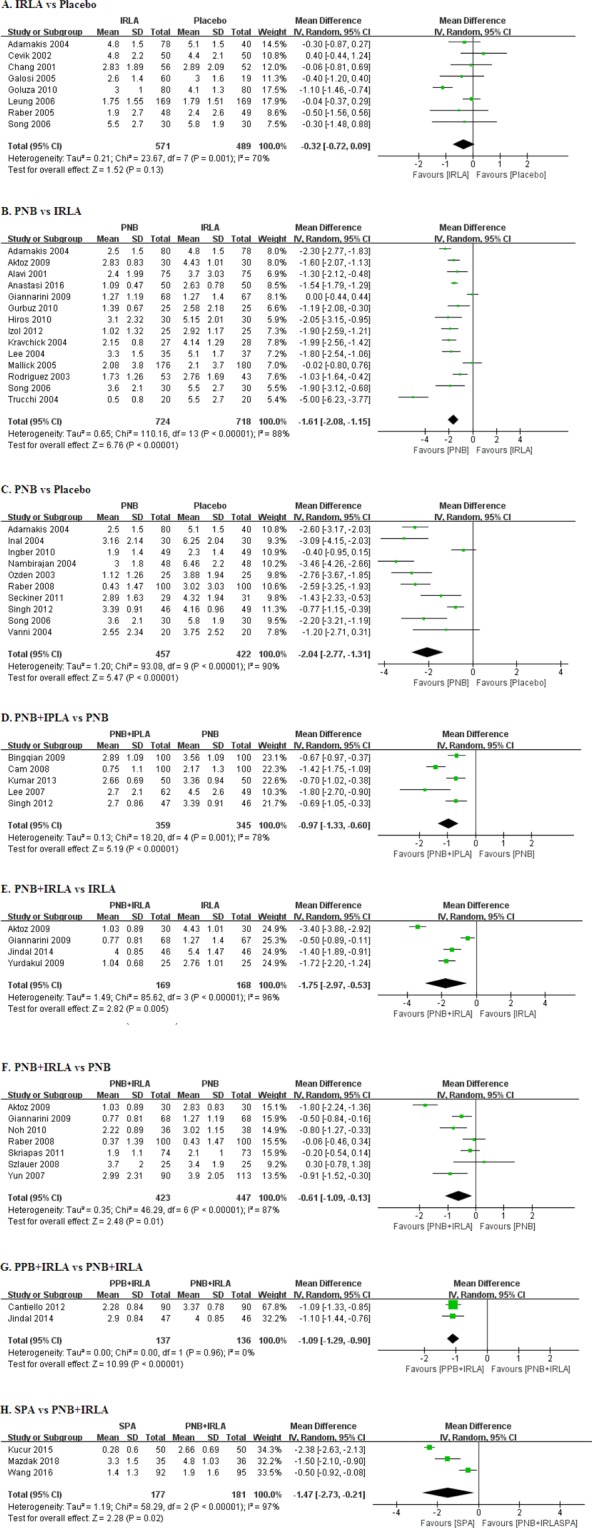
(**A**) Forest plots comparing IRLA with placebo, (**B**) Forest plots comparing PNB with IRLA, (**C**) Forest plots comparing PNB with Placebo, (**D**) Forest plots comparing PNB + IPLA with PNB, (**E**) Forest plots comparing PNB + IRLA with IRLA, (**F**) Forest plots comparing PNB + IRLA with PNB, (**G**) Forest plots comparing PPB + IRLA with PNB + IRLA, (**H**) Forest plots comparing SPA with PNB + IRLA IPLA: intraprostatic local anesthesia, IRLA: intrarectal local anesthesia, PNB: periprostatic nerve block, PPB: pelvic plexus block, SPA: spinal anesthesia.

Eight studies showed that IRLA demonstrated no statistically significant pain relief compared to placebo (MD: −0.32, 95% CI: −0.72 to 0.09, P = 0.13, Fig. 2A). Heterogeneity among the included studies was observed (P = 0.001; I^2^ = 70%).

#### PNB vs. IRLA

Fourteen studies showed that PNB was significantly lower compared to that of IRLA (MD: −1.61, 95% CI: −2.08 to −1.15, P < 0.00001, Fig. 2B). Heterogeneity among the included studies was observed (P < 0.00001; I^2^ = 88%).

#### PNB vs. Placebo

Ten studies showed that PNB significantly reduced pain compared with placebo (MD: −2.04, 95% CI: −2.77 to −1.31, P < 0.00001, Fig. 2C). Heterogeneity among the included studies was observed (P < 0.00001; I^2^ = 90%).

#### PNB + IPLA vs. PNB

Five studies showed that PNB + IPLA significantly reduced pain compared with PNB (MD: −0.97, 95% CI: −1.33 to −0.60, P < 0.00001; Fig. 2D). Heterogeneity among the included studies was not observed (P = 0.001, I^2^ = 78%).

#### PNB + IRLA vs. IRLA

Four studies showed that PNB + IRLA significantly reduced pain compared with IRLA (MD: −1.75, 95% CI: −2.97 to −0.53, P = 0.005; Fig. 2E). Heterogeneity among the included studies was observed (P < 0.00001; I^2^ = 96%).

#### PNB + IRLA vs. PNB

Seven studies were included in the analysis that compared PNB + IRLA with PNB (Fig. 2F). Combined PNB + IRLA resulted in significantly lower VAS scores than PNB alone (MD: −0.61, 95% CI: −1.09 to −0.13, P = 0.01). Heterogeneity among the included studies was observed (P < 0.00001; I^2^ = 87%).

#### PPB + IRLA vs. PNB + IRLA

Two studies showed that PPB + IRLA significantly reduced pain compared with PNB + IRLA (MD: −1.09, 95% CI: −1.29 to −0.90, P < 0.00001; Fig. 2G). Heterogeneity among the included studies was not observed (P = 0.96; I^2^ = 0%).

#### SPA vs. PNB + IRLA

Three studies showed that although SPA seems to have reduced pain compared with PNB + IRLA, the result was not statistically significant (MD: −1.47, 95% CI: −2.73 to −0.21, P = 0.02; Fig. 2H). Heterogeneity among the included studies was observed (P < 0.00001; I^2^ = 97%).

### Bayesian framework network meta-analysis

**Figure 3:**
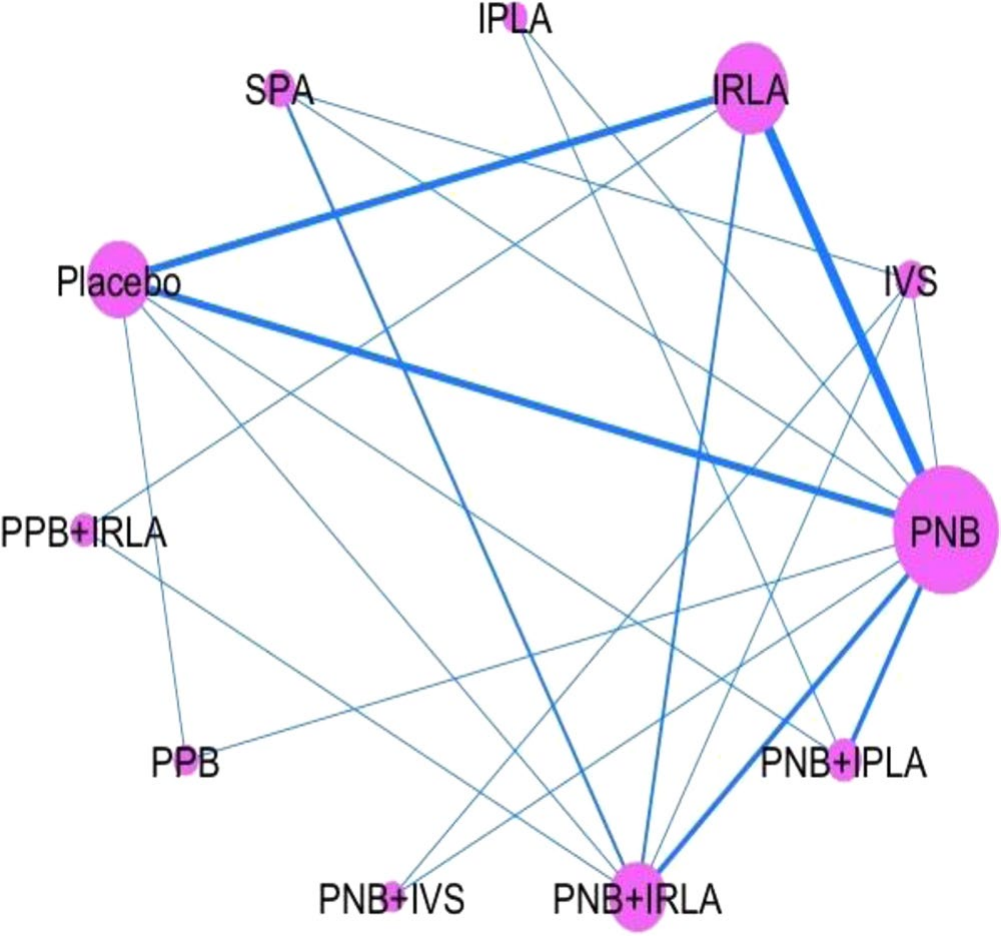
Network plot of evidence of all trials. The width of the lines is proportional to the number of trials comparing every pair of treatments, and the size of every node is proportional to the number of randomized participants. IPLA: intraprostatic local anesthesia, IRLA: intrarectal local anesthesia, IVS: intravenous sedation, PNB: periprostatic nerve block, PPB: pelvic plexus block, SPA: spinal anesthesia.

The network meta-analysis was performed with 47 studies. Eleven nodes (10 local anesthetic methods plus placebo) and 21 comparisons in the network plot of evidence are shown in Fig. 3. The width of each line is proportional to the number of trials comparing every pair of treatments, and the size of every node is proportional to the number of randomized participants.

**Figure 4:**
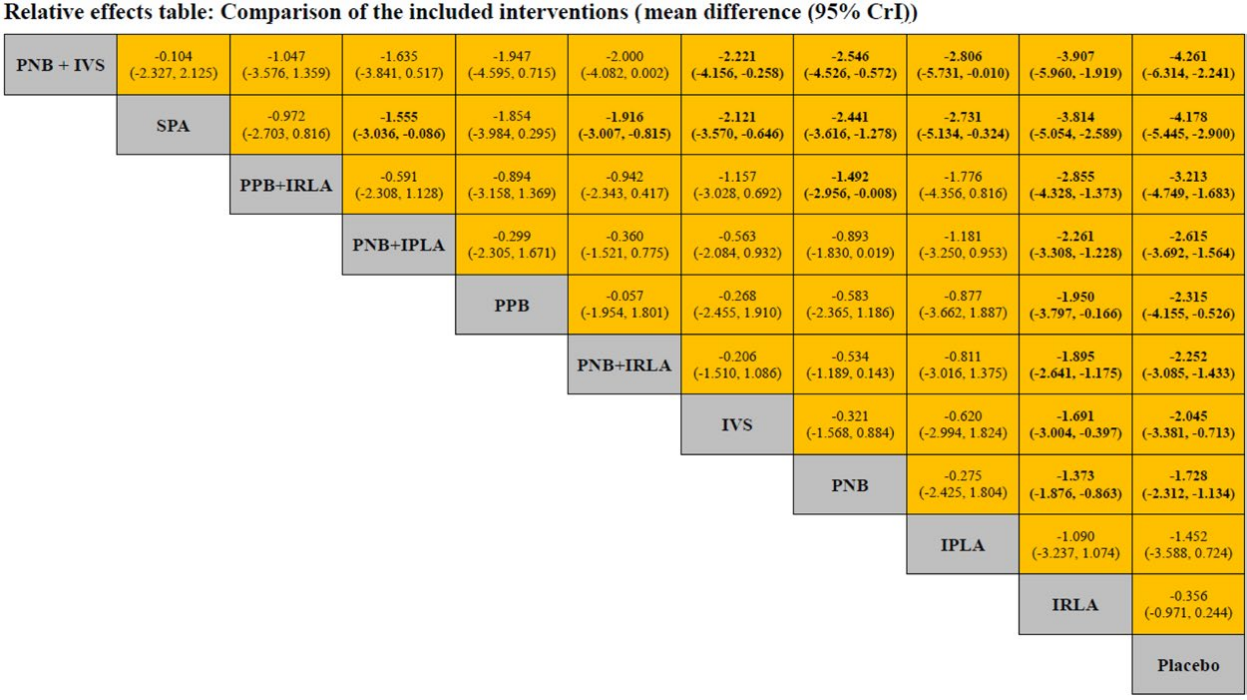
Relative effect table of local anesthetic method’s efficacy for pain control related prostate biopsy. Comparisons between treatments should be read from left to right, and the estimate is in the cell in common between the column-defining treatment and the row-defining treatment. For efficacy in local anesthesia, mean differences (MDs) less than 0 favor the column-defining treatment. Bold indicates statistical significance. IPLA: intraprostatic local anesthesia, IRLA: intrarectal local anesthesia, IVS: intravenous sedation, PNB: periprostatic nerve block, PPB: pelvic plexus block, SPA: spinal anesthesia.

**Figure 5:**
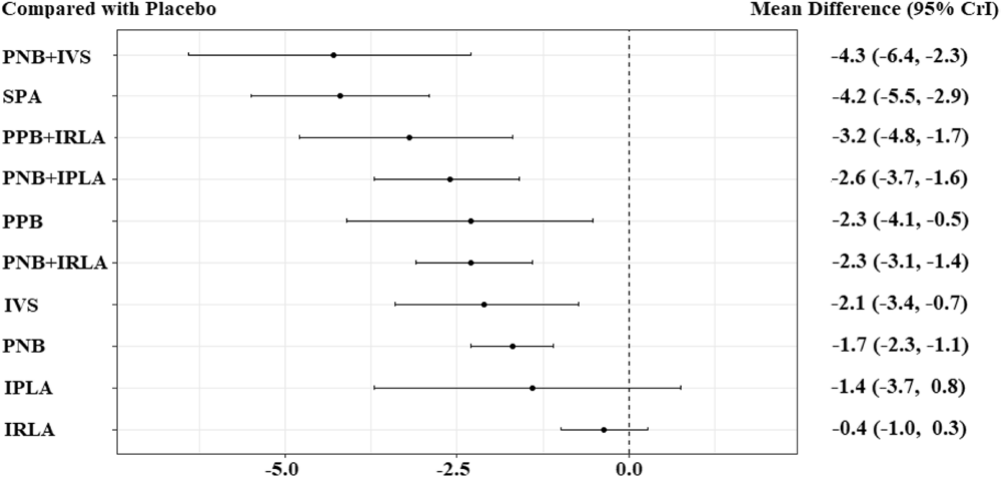
Relative effect plot of local anesthetic method’s efficacy for pain control related prostate biopsy IPLA: intraprostatic local anesthesia, IRLA: intrarectal local anesthesia, IVS: intravenous sedation, PNB: periprostatic nerve block, PPB: pelvic plexus block, SPA: spinal anesthesia.

**Figure 6:**
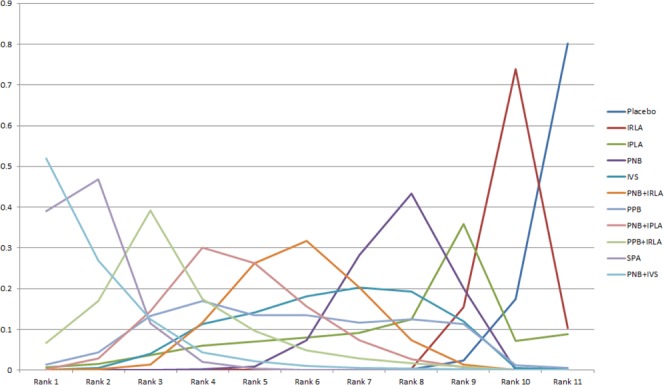
Rank probabilities plot for local anesthesia network of efficacy for pain control related prostate biopsy IPLA: intraprostatic local anesthesia, IRLA: intrarectal local anesthesia, IVS: intravenous sedation, PNB: periprostatic nerve block, PPB: pelvic plexus block, SPA: spinal anesthesia.

The network meta-analysis results are described in Fig. 4. Compared to placebo, PNB + IVS, SPA, PPB + IRLA, PNB + IPLA, PPB, PNB + IRLA, IVS, and PNB were significantly more effective on PBx-related pain control. Neither IPLA nor IRLA showed a significant difference in VAS compared to placebo. The relative effect plot confirmed these findings (Fig. 5). The rankings of the local anesthetic methods are similarly presented in Fig. 6. PNB + IVS and SPA were ranked first and the second, respectively, followed by PPB + IRLA, PNB + IPLA, PPB, PNB + IRLA, IVS, PNB, IPLA, and IRLA. There were 15 node-splitting models. With the exception of two models (PNB vs. PPB and PPB vs. placebo), 95% CrIs of inconsistency factors included zero and P-values of > 0.05 for the comparison between direct and indirect effects in all other node-splitting models.

There were 15 node-splitting models. With the exception of two models (PNB vs. PPB and PPB vs. placebo), 95% CrIs of inconsistency factors included zero and P-values of > 0.05 for the comparison between direct and indirect effects in all other node-splitting models. There were no significant inconsistencies in results in this analysis.

### Quality assessment and qualitative risk of bias

**Figure 7:**
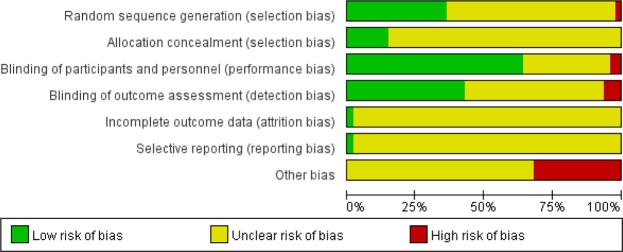
Risk of bias graph.

**Figure 8:**
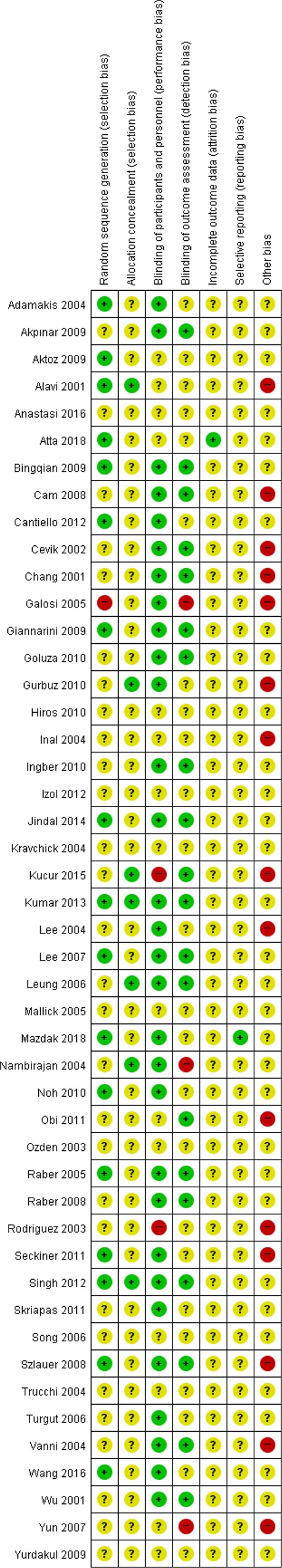
Risk of bias assessment. Green plus: low risk of bias, Yellow question: unclear risk of bias, Red minus: high risk of bias.

The risk of bias graph and assessment are summarized in Figs 7 and 8. There are three main sources of bias in the included trials. The first is insufficient number of participants in some trials, which may make it difficult to demonstrate the effect of local anesthesia. The second is that the number of biopsies taken was not identical for every patient in the trials. This variable may also affect the VAS score.

The results of GRADE quality assessment of direct evidence of each pairwise treatment comparison are shown in Table 2. Of the eight comparisons, certainty was low in six and very low in two.

**Table 2:** GRADE quality assessment of direct evidence of each pairwise treatment comparison.

Certainty assessment							Number of patients		Effect	Certainty	Importance
Number of studies	Study design	Risk of bias	Inconsistency	Indirectness	Imprecision	Other considerations	The former	The latter	Absolute (95% CI)		
**1. IRLA vs Placebo**											
8	randomized trials	serious^a^	not serious	not serious	serious^b^	none	571	489	MD **0.32 lower** (0.72 lower to 0.09 higher)	●●○○ LOW	CRITICAL
**2. PNB vs IRLA**											
14	randomized trials	serious^a^	serious^c^	not serious	not serious	publication bias strongly suspected^d^	724	718	MD **1.61 lower** (2.08 lower to 1.15 lower)	●○○ VERY LOW	CRITICAL
**3. PNB vs Placebo**											
10	randomized trials	serious^a^	serious^c^	not serious	not serious	none	457	422	MD **2.04 lower** (2.77 lower to 1.31 lower)	●●○○ LOW	CRITICAL
**4. PNB + IPLA vs PNB**											
5	randomized trials	serious^a^	serious^c^	not serious	not serious	none	359	345	MD **0.97 lower** (1.33 lower to 0.6 lower)	●●○○ LOW	CRITICAL
**5. PNB + IRLA vs IRLA**											
4	randomized trials	serious^a^	serious^c^	not serious	not serious	none	169	168	MD **1.75 lower** (2.97 lower to 0.53 lower)	●●○○ LOW	IMPORTANT
**6. PNB + IRLA vs PNB**											
7	randomized trials	serious^a^	serious^c^	not serious	not serious	none	423	447	MD **0.61 lower** (1.09 lower to 0.13 lower)	●●○○ LOW	IMPORTANT
**7. PPB + IRLA vs PNB + IRLA**											
2	randomized trials	serious^a^	not serious	not serious	serious^e^	none	137	136	MD **1.09 lower** (1.29 lower to 0.9 lower)	●●○○ LOW	IMPORTANT
**8. SPA vs PNB + IRLA**											
3	randomized trials	serious^a^	serious^c^	not serious	serious^e^	none	177	181	MD **1.47 lower** (2.73 lower to 0.21 lower)	●○○○ VERY LOW	IMPORTANT

**CI:** confidence interval, **IPLA**: intraprostatic local anesthesia, **IRLA**: intrarectal local anesthesia, **PNB**: periprostatic nerve block, **PPB**: pelvic plexus block, **MD:** mean difference, **SPA**: spinal anesthesia

**Explanations**.

^a^The risk of bias is most of unclear domains.

^b^The upper and lower limits of 95% CI include both meaningful benefit and harm.

^c^Significant heterogeneity observed.

^d^Funnel plot show significant asymmetry. Egger’s tests were less than 0.05 (P = 0.0002).

^e^Total number of participants is small.

## Discussion

PCa is the most frequently diagnosed cancer worldwide in men, with approximately 1.1 million new cases being diagnosed each year^51^. Assuming that the current PBx standard of using 10–14 biopsy cores detects prostate cancer in up to 44% of patients^52^, then PBx can be expected to be performed in approximately 2.5 million cases a year worldwide. Recently, various types of local anesthetic methods have been proposed to reduce the discomfort and pain associated with PBx^22^. Consequently, there is ongoing research to identify the perfect method for reduction of pain. Therefore, we performed a systematic review and network meta-analysis of RCTs published to date to determine the most effective anesthetic methods for pain control. Although similar meta-analyses^6,7,53–56^ have been performed previously, a new analysis was needed to incorporate the results of new trials that have been published on this topic. Moreover, previous meta-analyses only made direct comparisons, whereas we included multiple comparisons with more local anesthetic techniques (specifically, IVS, SPA, and PPB). Our study was an arm-based network meta-analysis with 11 arms (IPLA, IRLA, IVS, PNB, PPB, SPA, PNB + IPLA, PNB + IRLA, PNB + IVS, PPB + IRLA, and placebo).

The most commonly used local anesthetic methods for PBx are IRLA and PNB. National Comprehensive Cancer Network guidelines state that local lidocaine injection is more efficacious in reducing pain during probe insertion, whereas PNB reduces pain during the biopsy itself^57^. European Urological Association guidelines recommend PNB as the standard of care^58^. IRLA is a convenient local anesthesia method that causes only a little discomfort to patients^54^. The most commonly used agent is lidocaine alone or in combination with other molecules (prilocaine, nifedipine, dimethyl sulfoxide, etc.)^59^. Our results show that IRLA with lidocaine gel alone or in combination with other molecules could not significantly reduce the pain during the PBx, which is consistent with results from previous meta-analyses^7,53,54^. IRLA alone was the worst ranked method in this analysis other than placebo. Nash *et al*. were the first to describe the use of PNB before PBx in 1996^8^. Subsequently, many trials^2,9,12,21,22,25,28,30,34,36,37,40,42^ and meta-analyses^7,54,55^ have suggested that injection of local anesthetic around the nerve bundles might provide the best pain control during PBx. The results of our study also show that PNB significantly reduced pain compared with IRLA and placebo and are consistent with results from previous meta-analyses^7,54–56^. However, PNB alone had a probability of being ranked just seventh in effectiveness in the current study.

Combining PNB with IPLA or with IRLA results in a more extensive pain control effect^53^. In 2005, Mutaguchi *et al*.^60^ reported IPLA was a new local anesthesia technique for anesthetizing the prostate by blocking all sensory nerves from the posterior and anterior. Our results show that PNB with either IPLA or IRLA significantly reduced pain compared with placebo and had intermediate probability of being ranked fourth and sixth, respectively. However, IPLA alone did not significantly reduce the pain during the PBx. Recently, IPLA was shown to extend the time of PBx, but also provide improved pain control compared to PNB^59^. Nonetheless, more RCTs are required to justify the single use of IPLA in general.

Three recent trials investigated the effect of IVS for pain control during PBx^14,31,50^. Atta *et al*.^14^ conducted IVS by slow infiltration (3–5 min) of diazepam (5 mg) just before probe insertion and found that this IVS approach significantly decreases discomfort and fear of insertion of the probe and alleviates patient anxiety. In addition, PNB + IVS displays a significantly lower VAS score during PBx. Mazdak *et al*.^31^ performed IVS by infiltration of midazolam (25 μg/kg), fentanyl (2 μg/kg), and ketamine (1 mg/kg) through an antecubital vein at 5 min before taking the biopsies. They reported that the differences in VAS score between PNB + IRLA and IVS are not statistically significant. Additionally, they reported SPA significantly reduces pain compared with IVS. Turgut *et al*.^50^ injected midazolam (0.07–0.1 mg/kg) intravenously 5–10 min before the procedure. They showed that IVS displays a significantly lower VAS score compared with placebo, whereas no significant difference is observed between IVS and PNB. Our results show that IVS alone had no pain control effects compared with PNB alone, whereas combined PNB + IVS significantly reduced pain compared with PNB + IRLA. PNB + IVS had the highest probability of being ranked first. Nevertheless, IVS has a risk of respiratory and cardiovascular depression, and the use of anesthetic staff has cost implications^50^. Only three trials were analyzed in the aforementioned studies, so more RCTs should be performed for more accurate analysis of the effect of IVS.

PPB is also used to control biopsy pain. Wu *et al*.^47^ first described PPB using 5 mL of 1% lidocaine directly lateral to the tip of the seminal vesicles under grey-scale ultrasonography guidance. They found that this method does not reduce biopsy-related pain. Conversely, Akpinar *et al*.^10^ conducted a trial comparing PPB (using 2 mL of 2% lidocaine), to PNB at the same dosage and concentration. The authors reported that PPB provides superior analgesia to PNB because it acts on the prostate proximally. They suggested that the reasons for their divergent results are not clear but may be caused by the use of Doppler ultrasonography and larger cohorts unlike the study of Wu *et al*.^47^. Color Doppler ultrasonography is helpful for identifying the entire pelvic plexus with no influence of the left lateral decubitus position^59^. Another two studies reported that PPB + IRLA has significantly more effective pain control than PNB + IRLA^17,24^. Although the currently available guidelines do not mention the use of PPB, our study suggests the possibility that PPB might be superior to PNB. This concept requires more RCTs to confirm this possibility. Moreover, this procedure should be conducted only by skilled practitioners under the guidance of color Doppler ultrasonography, a requirement that may represent a limitation for this method.

SPA is an ideal anesthetic for anorectal surgery^1^ and had a highly probability of being ranked second in our study. Four of the included trials investigated the PBx pain control effect of SPA^1,31,34,46^. We classified the caudal block in one trial^46^ into one kind of SPA in which caudal block was accomplished using 20 mL of 1.2% lidocaine. Other trials used SPA with a low dose of 0.5% bupivacaine (0.5–1.5 mL) at the L3-5 level. They reported SPA significantly decreases pain during the procedure^1,31,34,46^. Moreover, there was no significant difference in anesthesia time or pain due to anesthesia itself compared with PNB. Nonetheless, SPA requires the presence of an anesthesiologist, which adds to the overall cost of the procedure and reduces the likelihood of SPA being practiced in an outpatient setting. Other drawbacks to SPA include a potential to cause a spinal headache or backache, systemic blood pressure or heart rate changes, and risk of infection into the subarachnoid space^1^.

To the best of our knowledge, ours is the first network meta-analysis to evaluate the optimal local anesthetic for controlling PBx-related pain in order to help clinicians select an appropriate anesthetic method for patients undergoing PBx. Our meta-analysis results suggest that various local anesthetic modalities, alone or in combination, were significantly more effective than placebo in alleviating pain for TRUS-guided PBx. Of these methods, PNB + IVS and SPA seem to be the most effective way to control PBx-related pain. However, several medical and socioeconomic factors should be considered because SPA and IVS are undoubtedly associated with increases of medical cost, need for anesthesiologist, need for extra medical personnel for monitoring patients’ recovery, longer hospital stay, and additional risk of morbidities, etc. In the current outpatient setting, PPB + IRLA, PNB + IPLA, PPB, PNB + IRLA, and PNB are acceptable modalities, but it is still uncertain whether PPB can be superior to PNB. Using the results of this study, clinicians could select the most effective method by providing a choice of anesthetic methods and discussing the methods with patients based on the patient’s personality, medical condition, and financial status, and the capabilities of the hospital.

We performed only the GRADE assessment of the direct comparison without assessing of indirect evidences. However, there are the GRADE Working Group approach for accessing the quality of treatment effect estimates from network meta-analysis^61^. Salanti *et al*. also suggested an approach to determining confidence in the output of a network meta-analysis^62^. They have emphasized the importance of assessing the quality and confidence of each pairwise comparison (containing direct, indirect or mixed evidence) and the ranking estimates. The judgments of the evidence of the network meta-analysis may differ from those of the evidence of the pairwise meta-analysis. Since most of the risk of bias for included studies was unclear, all of the GRADE risk-of-bias items were assessed as serious. This resulted in low and very low quality of all evidence due to the low quality of the studies included. Low level of GRADE assessment means that further research is highly likely to have a significant impact on our confidence in estimating effects and changes in estimates. Very low of GRADE assessment means that estimates of effects are highly uncertain. There was a discrepancy in the placebo arm among the included studies. For examples, some trials^3,23,32,35^ performed periprostatic saline injection, but others^5,9,18,19,29,37^ instilled placebo solutions into the rectum. Moreover, there was also no agreement in the type or dose of anesthetic in the same group. A major practical disadvantage of the VAS is that subsequent distance measurements are required^63^. Patients have difficulty finding the point that best describes the pain they feel. In other words, the VAS can be measured higher than actually talking about pain in words^64,65^. It may be easy to interpret the category of modest VAS meaningfully. Although VAS is may useful for the comparison of pain between the two groups, the interpretation of a single VAS may be subjective^63^. Network meta-analysis assumes that some treatment arms can be grouped in a similarity of rationality and procedure. Although there is added potential for combining studies that are not adequately similar, this assumption allows us to group treatment arms together as one node in the network^66^. Further high-quality RCTs are needed to overcome these drawbacks, to validate this result, and to find the optimal anesthetic method for PBx.

In conclusion, our meta-analysis suggests that various local anesthetic method, alone or in combination, were more effective in alleviating pain for TRUS-guided PBx than placebo. Of these anesthetic methods, PNB + IVS and SPA seem to be the most effective way to control PBx-related pain. Unfortunately, increase of medical cost and additional risk of morbidity must be considered before using these methods. In the current outpatient setting, PPB + IRLA, PNB + IPLA, PPB, PNB + IRLA, and PNB are the more acceptable options. Although the currently available guidelines do not mention the use of PPB, our study suggests that PPB might be a promising alternative to PNB. Further high-quality RCTs are needed to validate these results and clarify the optimal anesthetic method.

## Material and Method

This systematic review was registered in PROSPERO (CRD42018092602).

### Literature search

We performed literature searches on PubMed/Medline, Embase, Cochrane Library, Web of science, Scopus, and Clinical Key up to March 2018. Only published trials in English were included. Conference and meeting abstracts were excluded even if they met the eligibility criteria. The search terms used included ‘transrectal’, ‘prostate’, ‘biopsy’, ‘intraprostatic’, ‘periprostatic’, ‘intrarectal’, ‘spinal’, ‘anesthesia’, ‘block’, ‘pelvic plexus’, and relevant variants. A total of 1842 possible articles, of which 982 articles remained after removing duplicates, were found using these literature searches. Two authors (DKK and YSH) reviewed the titles and abstracts in consideration of the inclusion criteria and reviewed the independently identified papers. In cases of disagreement, the reviewers decided by agreement on whether or not to include the article.

### Trial inclusion criteria and exclusion criteria

The eligibility of a study was evaluated by the PICOS (participants, interventions, comparators, outcomes, and study design) approach and the Preferred Reporting Items for Systematic Reviews and Meta-Analyses guidelines^67^. Study population was defined as patients who underwent TRUS-guided PBx, and intervention was defined as local anesthesia. The comparator was defined as placebo or anesthetic method. The outcomes were biopsy pain scores that were measured using a visual analogue scale (VAS). Inclusion criteria were as follows: (1) RCT, (2) human research, (3) patients underwent TRUS-guided PBx, (4) use of local anesthesia, and (5) reported outcome values (VAS: mean and standard deviation could be calculated).

### Data extraction

Two independent authors (DKK and YSH) extracted the data using a predesigned form. Any conflicts in extracted data between the two authors were resolved via consensus. Extracted data included the first author, publication year, study design, treatment arms, number of patients, local anesthetic method and anesthetics, inclusion and exclusion criteria, number of PBx core, type of pain scale, and outcome. The main outcome was the biopsy pain score measured by VAS.

### Study quality assessments and quality of evidence

The risk of bias was evaluated in individual studies using tools recommended in recent meta-analysis guidelines that rate various aspects of RCT design and implementation^68^. Risk of bias was assessed for random sequence generation, allocation concealment, blinding of participants and researchers, blinding of outcome assessment, incomplete outcome data, selective reporting, and others.

The Grading of Recommendations, Assessments, Developments, and Evaluation (GRADE) was used to provide a systematic approach to the evaluation of the quality of evidence and strength of recommendations^69^. Criteria for consideration included assessment of methodology, precision and consistency of results, directness, and risk of publication bias. Based on five criteria, we assessed only direct evidence of pairwise meta-analysis by classifying the quality of evidence as one of four levels (i.e., high, moderate, low, and very low).

### Statistical analysis

A pairwise meta-analysis was performed on the comparison of a minimum of two studies. Outcomes are reported as a combination of the weighted mean difference (MD) with 95% confidence interval (CI) and the P-value. The pooled MD with 95% CIs expresses the difference in the size of the intervention effect. Statistical heterogeneity between trials was evaluated by Chi-square heterogeneity tests. The I^2^ statistic was also calculated to measure discrepancies between clinical trials. Either a P-value of < 0.05 for the Cochran Q statistic or an I^2^ statistic of > 50% indicated significant heterogeneity between trials^68^.

To indirectly compare the effect of each local anesthetic method on the endpoint (VAS), we conducted a network meta-analysis using a Bayesian hierarchical random effects model for continuous outcomes. This model estimates treatment-specific effects and effect differences. Pooled estimates were obtained using the Markov Chains Monte Carlo method in which each chain has 20,000 simulations and the first 5,000 simulations are discarded as burn-in. We modeled the continuous outcomes for every local anesthetic method of all trials and quantified the association between MDs with 95% credible intervals (CrIs) among studies (CrIs can be regarded as similar to conventional CIs). The selection of a random effects model for reported outcomes was based on the deviance information criteria. The random effects model is a measure of model fit that penalizes model complexity^70^. The node-splitting method was applied for computing the inconsistency of the model. The results of node-splitting analysis are considered to show no significant inconsistency when 95% CIs of inconsistency factors include zero or when there is a large probability value (P-value > 0.05) for the comparison between direct and indirect effects^71^. The relative effects were also assessed visually using the relative effects table and plots. Probability values were summarized and are reported as a rank probabilities table and plot.

We conducted the pairwise meta-analysis using Review Manager v.5.1 (The Nordic Cochrane Center, The Cochrane Collaboration, Copenhagen, Denmark, 2008). The network meta-analyses were conducted using R 3.4.3 (R development Core Team, Vienna, http://www.R-project.org) with the ‘GEMTC’ packages.

## Supplementary information

PRISMA NMA Checklist of Items to Include When Reporting A Systematic Review Involving a Network Meta-analysis
